# Validity of Multisensor Array for Measuring Energy Expenditure of an Activity Bout in Early Stroke Survivors

**DOI:** 10.1155/2018/9134547

**Published:** 2018-03-07

**Authors:** Sharon Flora Kramer, Liam Johnson, Julie Bernhardt, Toby Cumming

**Affiliations:** ^1^Stroke Division, Florey Department, The Florey Institute of Neuroscience and Mental Health, University of Melbourne, Melbourne, VIC, Australia; ^2^NHMRC Centre of Research Excellence in Stroke Rehabilitation and Brain Recovery, The Florey Institute of Neuroscience and Mental Health, Melbourne, VIC, Australia; ^3^Institute of Sport, Exercise and Active Living (ISEAL), Victoria University, Melbourne, VIC, Australia

## Abstract

*Introduction. *Stroke survivors use more energy than healthy people during activities such as walking, which has consequences for the way exercise is prescribed for stroke survivors. There is a need for wearable device that can validly measure energy expenditure (EE) of activity to inform exercise prescription early after stroke. We aimed to determine the validity and reliability of the SenseWear-Armband (SWA) to measure EE and step-counts during activity <1 month after stroke.* Materials and Methods. *EE was measured using the SWA and metabolic cart and steps-counts were measured using the SWA and direct observation. Based on walking ability, participants performed 2x six-minute walks or repeated sit-to-stands. Concurrent validity and test-retest reliability were determined by calculating intraclass and concordance correlation coefficients.* Results and Discussion. *Thirteen participants walked; nine performed sit-to-stands. Validity of the SWA measuring EE for both activities was poor (ICC/CCC < 0.40). The SWA overestimates EE during walking and underestimated EE during sit-to-stands. Test-retest agreement showed an ICC/CCC of <0.40 and >0.75 for walking and sit-to-stand, respectively. However, agreement levels changed with increasing EE levels (i.e., proportional bias). The SWA did not accurately measure step-counts.* Conclusion. *The SWA should be used with caution to measure EE of activity of mild to moderate stroke survivors <1 month after stroke.

## 1. Introduction

Cardiorespiratory fitness levels are low early after stroke (i.e., <1 month since stroke onset), with fitness levels of stroke survivors ranging from 44% to 76% that of age- and sex-matched sedentary healthy adults [1–3]. In stroke survivors, exercising at a moderate intensity, progressing to high intensity can elicit a cardiorespiratory training effect leading to improved cardiorespiratory fitness [4]. Our recent systematic review results showed that walking is more effortful for stroke survivors compared to healthy controls based on energy expenditure (EE) levels; and in some stroke survivors slow walking equals moderate intensity activity [5], which could lead to improvements in cardiovascular fitness. Understanding the EE of activities can inform development of exercise interventions in stroke survivors.

Indirect calorimetry using a metabolic cart is commonly used to measure the EE of physical activities. The metabolic cart measures the volume of oxygen uptake (VO_2_) using breath-by-breath analyses and has been shown to be a valid measure of VO_2_ uptake during different workloads in sedentary adults, moderately trained individuals, and athletes [6]. The development of mobile metabolic carts has extended the measurement from stationary activities (e.g., cycling on an ergometer or walking on a treadmill) to unfixed activities (e.g., overground walking). This more flexible method, while considered “gold standard,” remains cumbersome and costly and needs a trained staff member to operate the machine. It is not readily usable in an acute clinical stroke setting. Sensor-based technology, including wearable devices, may be a low-cost, noninvasive, and broadly applicable tool to measure the EE of physical activity in the clinic early after stroke, provided it is valid and reliable.

In a recent systematic review 60 different devices to measure physical activity in stroke were identified [7]. Of these devices, three had the potential to measure EE: the Actical (Koninklijke Philips NV), Body fixed sensor (Physilog, BioAGM), and the SenseWear Pro3 (Bodymedia Inc). The SenseWear (SWA) is worn on the upper arm and is the only multisensor device that includes accelerometer data as well as near body temperature, heat flux, and galvanic skin responses. Moore et al. showed that the SWA measure of daily EE, when worn on the unaffected arm (SWA_unaffected_), was highly correlated with doubly labelled water (gold standard) in chronic stroke survivors (2473 ± 468 versus 2380 ± 551 kcal/day, resp.) [8]. EE during walking measured by the SWA_unaffected_ showed* good* agreement (ICC > 0.70) and the SWA_affected_ showed* fair* agreement (ICC < 0.60) with a metabolic cart in chronic stroke survivors, suggesting that the SWA worn on the unaffected arm gives a more accurate estimate of EE during walking [9]. In the same study, the authors reported that the SWA step-count measures appear to be less promising [9]. It is unclear whether the easy-to-apply SWA device is a reliable and valid marker of EE in those with acute stroke. We wanted to determine the validity and reliability of the SWA_unaffected_ to measure EE during activity in people with acute stroke, that is, within the first month after stroke.

This study is a part of a larger study in which we aimed to compare EE of acute stroke survivors to healthy controls. In this paper we will only discuss the result regarding the validity and reliability of the SWA_unaffected_. Using a mobile metabolic cart as a gold standard we sought to determine the concurrent validity and reliability of SWA measures of EE during a physical activity in stroke survivors whose stroke onset was less than 1 month ago. We hypothesised that the SWA_unaffected_ is a valid and reliable tool to estimate EE during activity in acute stroke survivors.

## 2. Material and Methods

### 2.1. Participants

Stroke survivors admitted to the acute stroke ward at the Austin Hospital in Melbourne, Australia, were eligible to participate if they met the following criteria: (1) >18 years of age, (2) being within 1 month after stroke, (3) clinically diagnosed with stroke, (4) cognitively able to consent as assessed by the treating clinician, (5) sufficient English language command to follow complex instructions, and (6) medically cleared to participate by their treating clinician.

Stroke survivors were excluded from participating in the study if they had (1) comorbidities that impaired their ability to either walk or perform sit-to-stands (i.e., repeated standing up from and sitting down on a plinth) for six minutes (e.g., severe Chronic Obstructive Pulmonary Disorder, lower limb surgery) or (2) other neurological comorbidities that might affect the EE of activity (e.g., Parkinson's disease).

All stroke survivors who participated in this study provided written informed consent. The study was approved by the Austin Health Human Research Ethics Committee (reference number H2011/04447).

### 2.2. Assessment and Measurement

Each participant's age, height (measured using a stadiometer), weight while clothed but without shoes (measured using digital scales), and smoking status were recorded. Additionally, we obtained date of stroke, severity of stroke on admission, and stroke subtype from the participant's medical records. The National Institutes of Health Stroke Severity (NIHSS) scale was used to measure stroke severity with scores ranging from 0 to 42. Stroke severity was categorised into mild (<8), moderate (8–16), and severe (>16) [10].

### 2.3. Procedure

To measure EE of activity in both ambulatory and nonambulatory stroke survivors, we used two different test protocols: walking and sit-to-stand. Stroke survivors able to ambulate under supervision or with light manual support for balance and coordination (with or without a walking aid) performed the walking protocol. Stroke survivors who were unable to walk independently and needed manual support from at least two people performed the sit-to-stand protocol. We consulted with the participants' treating clinician (i.e., neurologist or allied health professional) to determine which protocol was most appropriate. All participants started with a three-minute resting period in a seated position followed by two bouts of six minutes of continuous activity (i.e., walking or sit-to-stands). Between the two bouts, participants rested for 30 minutes in different positions (i.e., seated, lying flat, and lying on an incline), allowing EE to return to baseline levels before commencing the second six-minute bout. We aimed for participants to reach steady-state during the six-minute activity bouts, with steady-state defined as variability in VO_2_ of less than 2.0 mLO_2_/kg/min over the last three minutes of activity [11].

Ambulatory stroke survivors walked back and forth along a 30-metre corridor at a self-selected pace. Participants performing the sit-to-stand protocol started in a seated position on a height-adjustable physiotherapy plinth, which was set at a height that required moderate effort and minimal assistance only for balance from the researchers. Before every test we emphasised to the participant that the goal was to move at a steady pace and* not* to move as fast as possible during the six minutes of activity.

EE was measured using a metabolic cart (Oxycon™ Mobile Device, CareFusion Australia Pty Ltd) and the SWA. For completeness, we applied one SWA on each arm (the arm most impaired from the stroke—*affected*—and the other arm—*unaffected*). We only reported and discussed data regarding the SWA_unaffected_; the data of the SWA_affected_ can be found in the supplementary materials.

The SWAs were placed dorsal on the upper limb midway between the elbow and shoulder joint. The SWA consists of triaxial accelerometers that record movement and position, and sensors that measure heat flux and galvanic skin response. Data from the accelerometers and sensors are integrated and converted to EE in Metabolic Equivalent of Tasks (METs) per minute using proprietary algorithms of the manufacturer.

The metabolic cart measured VO_2_ continuously using breath-by-breath analyses, averaged over three breaths; the readout of the data was in five-second epochs. Calibration of the metabolic cart was performed according to the manufacturer's operational instructions prior to testing. Gas calibration was performed against gas with a ratio of 16% O_2_ and 4% CO_2_ in Nitrogen. The ambient conditions were automatically measured by the unit. The participants were fitted with a facemask and a harness that carries the Oxycon units that allow telemetric transmission of data to a laptop.

Step-count was recorded by direct observation using a manual counter and by the SWA. The accelerometer output of the SWA is converted to step-counts using proprietary algorithms of the manufacturer. Sit-to-stand counts were also recorded by direct observation using a manual counter; a single sit-to-stand was counted when the participant stood up and sat back down.

The test protocol was terminated if a participant did not meet the following criteria assessed in a sitting position before the six-minute activity bouts: systolic blood pressure between 120 to 220 mm Hg (automatic blood pressure monitor OMRON, Australia), oxygen saturation > 92%, heart rate between 40 and 100 bpm (pulse oximeter, Oxycon Mobile Device, CareFusion Australia Pty Ltd), and temperature < 38.5°C (tympanic thermometer, Covidien, Medtronics, Australia). The test was also terminated if the participant requested stopping or felt unwell.

Purposeful sampling was used to recruit participants and we aimed to include stroke survivors who were able and not able to walk. We considered it feasible and practical to recruit 20 stroke survivors. We continued recruitment until 20 stroke survivors completed both activity bouts and had complete data sets including EE data measured by the metabolic cart and SWA_unaffected_.

### 2.4. Data Processing and Analysis

After the test had been completed the data of the metabolic cart and SWAs were downloaded on to a computer. In this study we expressed EE in METs. The Oxycon expresses EE in VO_2_ in ml/kg/min which is automatically converted to METs/min, where 1 MET is equal to a VO_2_ of 3.5 ml/kg/min.

The output of the metabolic cart data is averaged over five-second epochs; we averaged the metabolic cart data for each minute to match the SWA data, which is collected in METs/min. EE under steady-state conditions was calculated by averaging the EE output over the last three minutes of the six-minute bouts for each participant for both the data acquired by the metabolic cart and the SWAs. We used descriptive statistics, calculated medians and interquartile ranges (IQR) for demographic data, anthropometric data, walking speed, step-counts, and sit-to-stand counts, and we calculated means and standard deviations for the EE of steady-state activity.

We confirmed that all EE data were normally distributed using the Shapiro-Wilk test. We calculated intraclass correlation coefficients (ICC) and Lin's concordance correlation coefficients (CCC) to assess agreement between measures. Both ICC [12] and CCC [13] are valid statistics to determine agreement between measures.

Additionally we employed reduced major axis (RMA) regression, which is appropriate in the setting of this study, where both measurement tools produce readings that are susceptible to measurement error [14]. The regression analysis yields slope and intercept. A slope different from 1 is indicative of proportional bias. If the slope is 1 or close to 1 then the intercept needs to be interpreted, where an intercept different from 0 is indicative of fixed bias. The two components provide readings that differ by a consistent amount across magnitude (fixed bias) or that differ by a changing amount across magnitude (proportional bias) [15, 16]. We generated scatterplots including the line of perfect concordance and the RMA.

### 2.5. Outcomes

#### 2.5.1. Primary Outcome: Energy Expenditure

To test our hypotheses regarding* concurrent validity* of the SWA_unaffected_, we determined agreement between the metabolic cart and the SWA_unaffected_ for the following measures: (1) EE of steady-state walking and (2) EE of steady-state sit-to-stand.* Test-retest reliability* for both the metabolic cart and the SWA_unaffected_ was assessed by determining agreement between EE measurements taken during the 1st and 2nd bout during walking and sit-to-stand.

#### 2.5.2. Secondary Outcome: Step-Counts during Walking

Step-count measurements of the SWA utilise accelerometer data from the movement of the wearer's arm. When the wearer's arms are fixed on walking frames or supported by people walking with physical assistance, the recorded accelerometry data are likely to lack validity. We therefore excluded step-count data of participants who walked with a 4-wheel frame or required physical assistance. We tested our hypothesis regarding* concurrent validity* of the SWA by determining agreement between the observed step-counts and step-counts recorded by the SWA_unaffected_.* Test-retest reliability* of the SWAs was determined by agreement between the step-counts recorded during the 1st and 2nd bout of walking.

We used the following ICC and CCC cutoff points to interpret the strength of agreement: less than 0.40:* poor*, between 0.40 and 0.59:* fair*, between 0.60 and 0.74:* good*, and between 0.75 and 1.00:* excellent *[17]. All analyses were performed using Stata 13 (StataCorp LP).

## 3. Results

**Figure 1:**
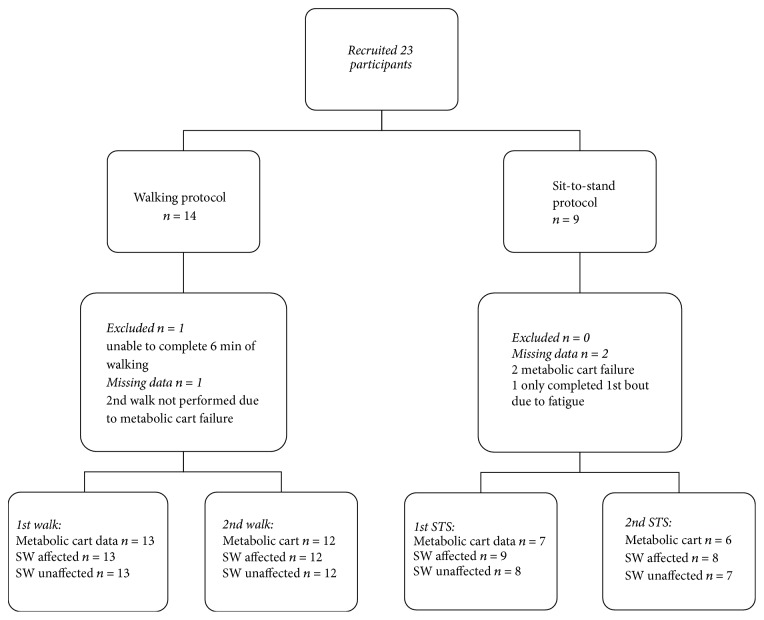
Flowchart of datasets for metabolic cart and SWA available for analyses.

We recruited 23 acute stroke survivors. Fourteen participants completed the walking protocol; one participant was unable to complete the second bout of walking due to fatigue. We excluded the data of another participant who completed less than 2.5 minutes of walking due to fatigue. Nine participants performed the sit-to-stand protocol; we had missing data for two participants due to machine failure and one participant was unable to perform the second bout of sit-to-stands due to fatigue. One participant had a bilateral stroke and performed the sit-to-stand protocol. The SWA data of both arms of this participant were regarded as EE of the affected arm (SWA_affected_). We included the average of EE values of both arms for this participant in the analyses; hence the difference in numbers of SWA measures in the group that performed sit-to-stand (Figure 1). The EE and step-count data of the SWA_affected_ compared to the metabolic cart and direct observations can be found in the supplement.

We analysed the data of 22 participants. The median age was 78 (IQR 73–81) and all participants performed the test within the first seven days after stroke (Table 1), except for one participant who was tested at 13 days after stroke. The median walking speed was 0.59 m/sec (IQR 0.54 to 0.72) and 0.60 m/sec (IQR 0.47 to 0.92) during the 1st and 2nd bout, respectively. Participants who performed both bouts of sit-to-stands were able to complete a median of 93 sit-to-stands (IQR 40 to 113) during the 1st bout (*n* = 7) and a marked higher median of 94 (IQR 40 to 121) during the 2nd bout (*n* = 7).

### 3.1. Concurrent Validity: EE Measured by Metabolic Cart and SWA_unaffected_

#### 3.1.1. Walking

**Figure 2:**
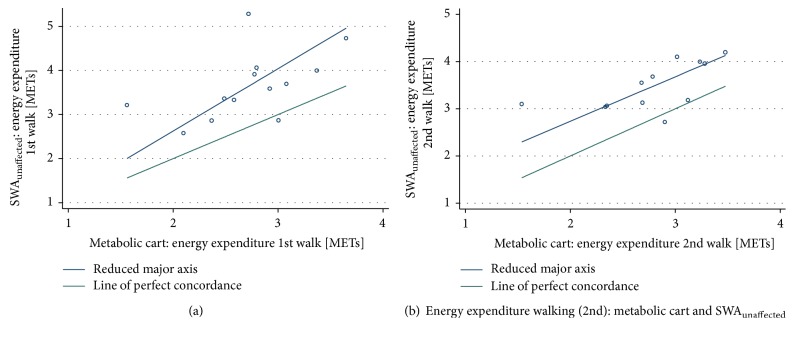


During both the 1st and 2nd bouts of walking we found* poor *agreement (ICC/CCC < 0.40) between EE measured by the metabolic cart and the SWA_unaffected_. There was evidence of proportional bias (slope = 1.42) during the 1st bout with agreement decreasing at higher levels of EE (Table 2 and Figure 2(a)), whereas the slope during the 2nd bout was 0.94; the SWA_unaffected_ systematically overestimated EE (Table 2 and Figure 2(b)).

#### 3.1.2. Sit-to-Stands

We found* poor* agreement (ICC/CCC < 0.40) between the EE of sit-to-stands measured by the metabolic cart and the SWA_unaffected_ for both sit-to-stand bouts. During the 1st bout of sit-to-stands, there was little evidence for proportional bias; the SWA systematically underestimated EE (intercept = −0.57). During the 2nd bout there was some evidence of proportional bias (slope = 0.64) where agreement decreased at higher EE levels (see Table 2).

### 3.2. Test-Retest Reliability: EE during Walking

We first assessed the test-retest reliability of our gold standard, the metabolic cart, and found excellent agreement between the EE measured during the 1st and the 2nd bout of walking (ICC = 0.96, 95% CI 0.87 to 0.99; CCC = 0.96, 95% CI 0.90 to 1.00, *n* = 12). There was no evidence of proportional or fixed bias (slope = 1.05 and intercept = −0.19) indicating that participants performed at a similar level across bouts.

**Figure 3:**
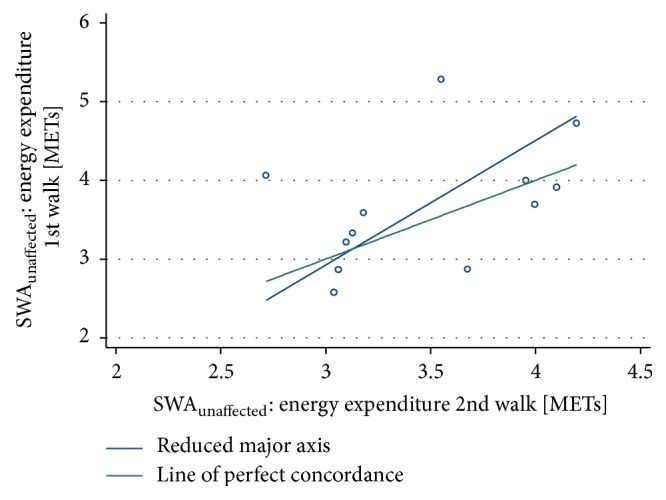
SWA_unaffected_: energy expenditure between 1st and 2nd bout of walking.

However, this was not the case for EE of walking measured using the SWA_unaffected_, which showed* poor *agreement between the two time points (ICC = 0.39, 95% CI 0.0 to 0.77; CCC = 0.37, 95% CI −0.08 to 0.82, *n* = 12). The RMA showed proportional bias (slope = 0.63 and intercept = 1.15) indicating that when EE levels increase just beyond 3 METs, agreement gets poorer (Figure 3).

### 3.3. Test-Retest Reliability: EE during Sit-to-Stands

The metabolic cart was reliable between bouts of sit-to-stands, similar to our finding for walking, with* excellent* agreement of EE measures between the 1st and 2nd bout of sit-to-stands (ICC = 0.93, 95% CI 0.64 to 0.99; CCC = 0.92, 95% CI 0.82 to 1.0, *n* = 6); however, there was some evidence of proportional bias (slope = 0.74 and intercept = 0.53) with agreement reducing at higher levels of EE. In the scatterplot (not shown) we identified one clear outlier. This individual moved substantially faster during the second bout compared to the 1st bout, completing 139 sit-to-stands versus 116, respectively. Post hoc analysis of the data excluding the outlier resulted in an ICC of 0.99 (95% CI 0.93 to 1.00) and a CCC of 0.99 (95% CI 0.96 to 1.0; *n* = 5) and a slope and intercept of 1.12 and −0.25, respectively, indicating that the remaining participants performed at a similar rate between bouts.

**Figure 4:**
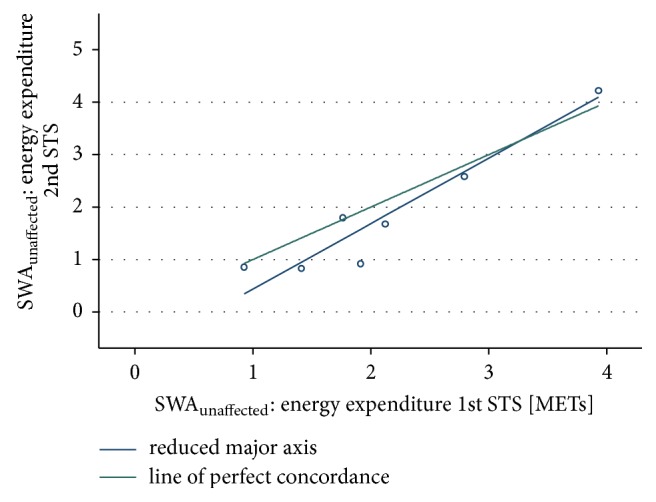
Energy expenditure SWA_unaffected_: 1st and 2nd bout of sit-to-stands.

Agreement was* excellent *for EE measures between the 1st and 2nd bout of sit-to-stands recorded by the SWA_unaffected_ (ICC = 0.90, 95% CI 0.58 to 0.98; CCC = 0.89, 95% CI 0.74 to 1.0, *n* = 7). The RMA showed proportional bias (slope = 0.80 and intercept = 0.65) with agreement improving up to an EE level of 3.5 (Figure 4).

### 3.4. Concurrent Validity: Step-Counts Measured by Observed Step-Counts and SWA_unaffected_

We excluded step-count data of five participants who walked with a walking frame or needed physical assistance; the analyses included data of 8 participants. The average number of step-counts measured by the SWA_unaffected_ was substantially lower (>190 steps) than the average step-counts measured via direct observation (Table 3).

The SWA_unaffected_ showed* poor* agreement with observed step-counts during the 1st bout and 2nd bout of walking and the RMA slopes were large showing evidence of proportional bias with agreement improving when step-counts increase (Table 3).

## 4. Discussion

We set out to determine if the SWA wearable device could provide a valid measure of EE during physical activity in acute stroke patients. Our results indicated that the SWA worn on the unaffected arm did not accurately measure EE during a bout of physical activity; rather, it seemed to overestimate EE compared to the metabolic cart during walking and underestimate EE during sit-to-stands. Our findings regarding EE during walking are in contrast with the findings in the Manns and Haennel (2012) study. Their study, which included 12 chronic stroke survivors, found* good* agreement (ICC = 0.70) between EE measured by the metabolic cart and the SWA during a six-minute walk [9]. In our study agreement was* poor* (CCC < 0.40). It has been suggested that slower walking speeds might lead to decreased accuracy of EE estimation by the SWA [9]. Our results do not support this suggestion, since the participants in our study walked at a higher average speed and used less energy compared to the participants in the Manns and Haennel study. The SWA uses an algorithm that integrates information collected from the triaxial accelerometer and the sensors that detect heat flux, temperature, and galvanic skin reaction, to estimate EE. There are no details available regarding this algorithm, preventing us from exploring which factors might have influenced the accuracy of EE estimates.

We found that the SWA greatly underestimated step-counts by approximately 300 steps on average within two weeks of stroke onset. This difference was larger than the difference found in the Manns and Haennel study in which step-counts measured by the SWA were compared to step-counts measured by the StepWatch Activity Monitor (SAM) in stroke survivors > 6 months after stroke [9]. The SAM is a highly accurate device to measure step-counts [18] and it is therefore unlikely that the use of a different criterion could be an explanation for the difference in agreement between our studies. Recall that step-counts recorded by the SWA are mostly derived from the accelerometer data which is based on arm swing. To minimise the impact of reduced arm swing, we purposefully only reported data from the SWA_unaffected_ and excluded data from participants that walked with physical assistance or with a walking aid from the step-count analyses. It is possible, though, that the included participants also had lower-than-normal arm swing during walking. The results of our study confirm that the SWA is not a valid tool to measure step-counts in stroke survivors. A recent review highlights that there are other accelerometer based tools available that validly and reliably measure step-counts [7].

It is however important that our results are interpreted with caution; no formal calculations were performed to predetermine sample size and the sample size of this study was small. On the other hand we did compare the data of the SWA to the metabolic cart, which is regarded as a gold standard, and showed that in our sample it was a reliable measure of EE of activity and we are confident that the metabolic cart is a true measure of EE. Including a broad variety of stroke survivors in a clinical study like this is challenging. We were able to include ambulatory and nonambulatory stroke survivors within 14 days of stroke onset. Almost all of our participants, except one, were able to reach steady-state (22/23), regardless of their ability to ambulate. Exercising under steady-state conditions is one of the methods to improve cardiorespiratory fitness and we showed that some stroke survivors have the potential to start performing cardiorespiratory exercise early after stroke. It is important, however, that all of the included stroke survivors had mild to moderate stroke severity.

The differences between SWA and metabolic cart were systematic and small. During walking, EE was overestimated by less than 1.0 MET and during sit-to-stands it was underestimated by less than 1.1 METs. Considering that the range of activity intensity is 3 METs, that is, light intensity is <3 METs and moderate intensity is 3–6 METs, a difference of less than 1.1 METs is relatively small. It is a concern, however, that the direction of the estimation error was not consistent across the two activities we tested. Therapy session in stroke rehabilitation can consist of different exercises including walking and sit-to-stand amongst other activities. Furthermore the test-retest reliability of the SWA_unaffected_ measuring EE showed mixed results regarding agreement, but most importantly the RMAs for both walking and sit-to-stands showed that agreement changed when levels of EE change. This suggests that SWA EE output during therapy sessions with mixed activities would be highly variable, and using the SWA would not be a reliable method to track EE over time.

## 5. Conclusion

Based on the results of our study the SWA does not accurately measure EE and therefore should be used with caution when measuring EE during activities early after stroke.

## Figures and Tables

**Table 1:** Participant characteristics.

	All (*n* = 22)	Walking (*n* = 13)	Sit-to-stand (*n* = 9)
Age, years	78 (70 to 83)	78 (70 to 85)	78 (73 to 78)
Male (*n*)	13	9	4
Time since stroke, days	4 (2 to 6)	4 (2 to 6)	4 (3 to 5)
Height, cm	164.5 (159.0 to 173.0)	163.0 (161.8 to 171.0)	165.0 (159.0 to 173.0)
Body weight, kg	73.0 (62 to 90)	72.2 (61.1 to 81.3)	76.7 (72.2 to 90.0)
Affected side, *n* = right/ bilateral	8/1	5/0	3/1
Stroke severity:			
Mild NIHSS < 8 (*n*)	18	11	7
Moderate NIHSS 8–16 (*n*)	4	2	2
Severe NIHSS > 16 (*n*)	0	0	0

All data is reported as medians and IQRs unless stated otherwise.

**Table 2:** Energy expenditure of walking and sit-to-stands and agreement between the metabolic cart and SWA_unaffected_.

Outcome energy expenditure (METs)							
Activity bout (*n*)	Metabolic cart	SWA_unaffected_	Mean difference (SD)	ICC (95% CI)	CCC (*r*_*c*_) (95% CI)	RMA slope	RMA intercept
1st walk (*n* = 13)	2.72 (0.54)	3.65 (0.76)	−0.93 (0.66)	0.02 (0.0 to 0.54)	0.24 (−0.02 to 0.51)	1.42	−0.21
2nd walk (*n* = 12)	2.78 (0.52)	3.47 (0.49)	−0.69 (0.45)	0.13 (0.0 to 0.63)	0.31 (0.02 to 0.61)^*∗*^	0.94	0.85
1st sit-to-stands (*n* = 6)	2.35 (0.95)	2.21 (0.94)	0.47 (0.79)	0.38 (0.0 to 0.88)	0.37 (−0.33 to 1.00)	1.04	−0.57
2nd sit-to-stands (*n* = 5)	2.49 (1.07)	1.83 (1.22)	1.08 (0.85)	0.25 (0.0 to 0.88)	0.34 (−0.19 to 0.86)	0.64	−0.14

Energy expenditure is reported as mean (SD); ^*∗*^*p* = 0.03; ICC: intraclass correlation coefficient; CCC: concordance correlation coefficient; RMA: reduced major axis.

**Table 3:** Step-counts and agreement between observed count and SWA_unaffected_.

Outcome step-counts							
Activity bout (*n*)	Observed counts	SWA_unaffected_	Mean difference (SD)	ICC (95% CI)	CCC (95% CI)	RMA slope	RMA intercept
1st walk (*n* = 8)	592 (87)	356 (219)	−235 (173)	0.0 (0.0 to 0.66)	0.22 (−0.04 to 0.48)	2.52	−1133
2nd walk (*n* = 8)	602 (87)	411 (207)	−191 (150)	0.16 (0.0 to 0.74)	0.30 (0.01 to 0.59)^*∗*^	2.39	−1026

All data is reported as mean (SD) unless stated otherwise; ^*∗*^*p* = 0.03; ICC: intraclass correlation coefficient; CCC: concordance correlation coefficient; RMA: reduced major axis.

## Supplementary Materials

Supplementary materials (1) EE and agreement between metabolic cart and SWA_affected_. (2) Step-count agreement between observed counts and SWA_affected_.
