# Supplementary material for: Validity of Multisensor Array for Measuring Energy Expenditure of an Activity Bout in Early Stroke Survivors
Source: Stroke Res Treat. 2018 Mar 7;2018:9134547. doi: 10.1155/2018/9134547 (PMC5863318; doi:10.1155/2018/9134547)
Supplement: Supplementary materials — (1) EE and agreement between metabolic cart and SWAaffected. (2) Step-count agreement between observed counts and SWAaffected. [file 9134547.f1.pdf]

Supplement

1 EE and agreement between metabolic cart and $SWA_{affected}$

**Outcome (n)**	**Metabolic cart**	**$SWA_{affected}$**	**ICC 95%CI**	**CCC (rc) 95%CI**	**Mean difference (SD)**	**RMA slope**	**RMA intercept**
EE 1st walk (n = 13)	2.27 (0.54)	3.28 (1.32)	0.42 (0.0 to 0.78	0.43 (0.18 to 0.69)	0.56 (1.01)	2.46	-3.42
EE 2nd walk (n = 12)	2.78 (0.53)	3.37 (0.90)	0.21 (0.0 to 0.68)	0.27 (-0.10 to 0.65)	0.56 (0.84)	-1.06	2.20
EE 1st STS (n = 7)	2.15 (0.57)	1.32 (0.56)	0.0 (0.0 to 0.72)	0.22 (-0.13 to 0.56)	-1.03 (0.63)	0.77	-0.49
EE 2nd STS (n = 6)	2.49 (1.07)	1.17 (0.42)	0.0 (0.0 to 0.76)	0.23 (-0.05 to 0.49)	-1.33 (0.74)	0.40	0.18

All data is reported as means (SD) unless stated otherwise. ICC=intraclass correlation coefficient, CCC=Lin's correlation coefficient, RMA=reduced major axis

2. Step-count agreement between observed counts and $SWA_{affected}$

Outcome (n)	Observed counts	$SWA_{affected}$	ICC (95%CI)	CCC (95%CI)	Mean difference (SD)	RMA slope	RMA Intercept
*Step-count 1st walk (n = 8)**	576 (544 to 624)	312 (123 to 547)	0.19 (0.0, 0.65)	0.19 (-0.45 to 0.42)	-267 (202)	2.84	-1360
Step-count 2nd walk (n = 8)	595 (553 to 646)	335 (168 to 609)	0.08 (0.0, 0.60)	0.24 (-0.02 to 0.50)	-232 (199)	2.91	-1382

All data are reported as medians (IQRs) unless stated otherwise. ICC=intraclass correlation coefficient, CCC=Lin's correlation coefficient, RMA=reduced major axis. *Analyses exclude n=5 participants who walked with a walking aid.
